# 

**Published:** 1894-03

**Authors:** 


### CONTENTS.

### COMMUNICATIONS.

Some Suggestions as to the Relation of the Teeth to Empyema of the Maxillary Sinus.......... 105
Systemic Medication in Dental Practice.......... 116
Abstract—Chairman's Address.......... 125
Some Points on the Articulation of Teeth.......... 127
A Plate Retainer.......... 143
Special Notice.......... 144

### SELECTIONS.

Squelching a Quack.......... 144
Importance of Dyspepsia.......... 145
A Table of Food Values.......... 147
Gastric Intestinal Affections.......... 148
Woman's Health Protective Association.......... 149
Superfluous Spectacles.......... 149

### NOTICES.

Illinois State Dental Society.......... 150
Alabama Dental Association.......... 151
American Medical Association.......... 151
The Transactions of the World's Columbian Dental Congress.......... 151

### EDITORIAL.

Care of the Mouth and Teeth.......... 152
The Tri-State Dental Meeting.......... 154
Bibliographical.......... 155
Dentistry in Court.......... 156

